# Neural Dynamics of the Combined Discounting of Delay and Probability During the Evaluation of a Delayed Risky Reward

**DOI:** 10.3389/fpsyg.2020.576460

**Published:** 2020-09-29

**Authors:** Guangrong Wang, Jianbiao Li, Shuaiqi Li, Chengkang Zhu

**Affiliations:** ^1^Neural Decision Science Laboratory, School of Economics and Management, Weifang University, Weifang, China; ^2^School of Economics, Institute for Study of Brain-Like Economics, Shandong University, Jinan, China; ^3^Department of Economic and Management, Nankai University Binhai College, Tianjin, China

**Keywords:** delay, probability, combined discounting, event-related brain potential, neural dynamics

## Abstract

Delay discounting and probability discounting are two important processes, but in daily life there are many more situations that involve delayed risky outcomes. Although neuroscience research has extensively investigated delay and probability discounting in isolation, little research has explored the neural correlates of the combined discounting of delay and probability. Using the event-related brain potentials (ERPs) technique, we designed a novel paradigm to investigate neural processes related to the combined discounting of delay and probability during the evaluation of a delayed risky reward. ERP results suggested distinct temporal dynamics for delay and probability processing during combined discounting. Both the early frontal P200 and the N2 reflected only probability, not delay, while the parietal P300 was sensitive to both probability and delay. Furthermore, the late positive potential (LPP) was sensitive to probability, but insensitive to delay. These results suggest that probability has a prolonged modulatory effect on reward evaluation in the information processing stream. These findings contribute to an understanding of the neural processes underlying the combined discounting of delay and probability. The limitation of this study is to only consider four delay and probability combinations. Future studies can explore the combined discounting of more probability and delay combinations to further test the robustness of the conclusion.

## Introduction

The subjective value of a reward is altered by its delay or likelihood. If a delay reduces the subjective value of a reward, then this tendency is labeled “delay discounting.” If the subjective value of a reward is altered by likelihood of obtaining it, then this tendency is labeled “probability discounting” (Frederick et al., 2002; McKerchar and Renda, 2012). Although inter-temporal decisions and risky decisions are common, delayed risky decisions are much more common in real life. For example, when making a financial investment, the possible gain pays off in the future. Many human social behaviors, like smoking, physical exercise, substance abuse, and education, often involve the simultaneous use of delay and probability discounting.

Although delay and probability discounting have been studied extensively, the majority of studies have investigated these two discounting processes in isolation; only a few studies have focused on the combined discounting of a delayed risky outcome.

Theoretical studies have provided different models that might be able to explain how delay and probability discounting combine. Killeen (2009) suggested an additive model whereby delay and probability discounting functions are combined additively, while other multiplicative models propose that these functions are combined in a multiplicative fashion (Ho et al., 1999; Cox and Dallery, 2016, 2018). An additive model posits that the effect of delay is independent of probability and vice versa. Conversely, a multiplicative model assumes that the effects of delay and probability are dependent each other. The interaction effect between delay and probability can be used to distinguish between additive and multiplicative models (Vanderveldt et al., 2015).

Recently, some behavioral studies have tried to examine the combination of delay and probability discounting. The results of Vanderveldt et al. (2015) showed a significant interaction between delay and probability factors, which is consistent with the multiplicative models. Shavit and Rosenboim (2015) distinguished between the effects of delay and probability. They suggested that when risky assets are delayed, both outcome and risk are delayed. Weatherly et al. (2015) revealed that delay discounting of a reward was changed by its likelihood, while probability discounting was almost unchanged by its delay. Taken together, their findings suggest that delay and probability discounting may differentially interact with one another and that the probability weighting is much larger than the delay weighting during the evaluation of a delayed risky outcome.

Extensive neuroscience research has also separately investigated delay discounting and probability discounting; however, to our knowledge, almost no neural research has focused on combined effects of these two discounting. Therefore, we tried to explore the neural correlates of processing a delayed risky reward using the event-related potentials (ERPs) technique.

Neuroscience research has demonstrated that a range of brain regions are related to delay discounting, such as medial prefrontal cortex, orbitofrontal cortex, ventral striatum, anterior insula, temporal-parietal cortex, dorsolateral prefrontal cortex, anterior cingulate cortex, posterior cingulate cortex, and lateral parietal cortex (Kable and Glimcher, 2007, 2010; Peters and Büchel, 2009, 2010; Carter et al., 2010; Liu and Feng, 2012; Liu et al., 2012; Hare et al., 2014). The ventromedial prefrontal cortex, anterior cingulate cortex, posterior cingulate cortex, striatum, amygdala, and insula are considered to be involved in probability discounting (Paulus and Frank, 2006; Berns et al., 2008; Hsu et al., 2009; Peters and Büchel, 2009; Smith et al., 2009; Polezzi et al., 2010; Berns and Bell, 2012; Blankenstein et al., 2018).

Previous ERP studies have also investigated the temporal courses of delay and probability discounting separately. These studies have identified several important ERP components that reflect the processing of delay and probability discounting. The frontal P200 was shown to represent the early valuation of time delay and probability discounting (Gui et al., 2016; Wang et al., 2019). The N2 was more negative following a long delay or low probability relative to a short delay or high probability (Wu and Zhou, 2009; Yang et al., 2015; Gui et al., 2016; Xia et al., 2017; Wang et al., 2019). The P300 was found to reflect probability discounting (Oberg et al., 2011; Wang et al., 2015) and delay discounting (He et al., 2012; Gui et al., 2016; Xia et al., 2017).

In this study, a delayed risky choice paradigm was designed to explore the neural correlates of the combined discounting of delay and probability. In our paradigm, participants were asked to select from an immediate certain option and a delayed risky option: the immediate certain option was set as 50 Chinese Yuan (CNY), and the delayed risky option was set as 100 CNY. By controlling for the effect of magnitude, we were able to investigate the neural correlates underlying the combined discounting of delay and probability.

Based on existing studies, several ERP components have been found to be related to delay and probability discounting. Therefore, we focused on these ERP responses that are associated with delay and probability during the evaluation of delayed risky rewards. Because the P200 component is related to quick assessment of a stimulus (Crowley and Colrain, 2004; Lau et al., 2013; Gui et al., 2016; Wang et al., 2019), we hypothesized that the P200 component would represent the delay and probability of delayed risky rewards. The N2 is considered to be sensitive to the early appraisal of time delay and probability and becomes more negative for bad outcomes relative to good outcomes (Hewig et al., 2007; He et al., 2012; Cherniawsky and Holroyd, 2013; Gui et al., 2016; Xia et al., 2017; Wang et al., 2019). In our paradigm, given the same probability, the short-delay rewards were considered to be better than the long-delay rewards. The similar conclusion can be made for probability. Hence, the hypothesis that the N2 would represent processes of both delay and probability was proposed. Moreover, the P300 component is considered to represent elaborative outcome evaluation and demonstrated to represent delay and probability discounting (Wu and Zhou, 2009; Righi et al., 2014; Gui et al., 2016; Wang et al., 2019). Therefore, we hypothesized that the P300 would encode both delay and probability processes.

## Materials and Methods

### Participants

Twenty-six right-handed undergraduates (12 females and 14 males) were recruited to participate in the experiment. The mean age was 21.11 years (SD = 1.31). All participants had normal or corrected-to-normal visual acuity and no history of neurological or mental disease. All participants signed an informed consent prior to the experiment, which was performed in accordance with the Declaration of Helsinki and was approved by the Ethics Committee of the School of Economics, Shandong University, China. The participants were informed that although rewards in current task were hypothetical, they would be rewarded handsomely only if they carried out the experiment carefully. Each participant was paid an average of 65 CNY (approximately $10).

The G*Power 3.1 was performed for sample size estimation (Faul et al., 2007). Considered to medium effect with a power of *β* = 0.80 and *α* level of 0.05, the required sample size is 24. Our proposed sample size of 26 will be more than adequate for the main objective of this study.

### Task and Stimuli

This study aimed to investigate neural dynamics of the combined discounting of probability and delay. Because the subjective value of a delayed risky option depends on its magnitude, delay, and probability, we used the following experimental designs to control for related factors. First, participants were told to select from an immediate certain option and a delayed risky option. Second, for each choice, the magnitude of immediate certain option was set as 50 CNY, and the magnitude of delayed risky option was set as 100 CNY; this allowed us to control for the effect of magnitude. Third, option and choice of each trial was displayed serially, and this allowed us to isolate the reward valuation process from selection process.

Because combined discounting of a delayed risky option is altered by its delay and probability, the experiment applied a 2 (delay) × 2 (probability) factor design, with the reward being either short delay (1 week or 2 weeks) or long delay (11 months or 12 months), and either low probability (20 or 30%) or high probability (80 or 90%). There were four conditions: short delay and low probability (SD + LP); short delay and high probability (SD + HP); long delay and low probability (LD + LP); and long delay and high probability (LD + HP). Each condition consisted of 48 trials.

### Procedure

Participants were instructed of the rules of the experimental task by explaining the written instructions. The task was performed in a quiet and isolated laboratory. The participants were told that they would be paid for participation after completing the experiment. The recording session took approximately 40 min.

**Figure 1:**
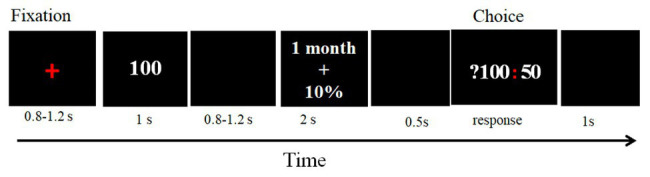
Sequence of trial events.

The participants first completed eight practice trials to understand the experimental task. A total of 192 test trials were randomly divided into four blocks with 48 trials each. Each trial began with a red cross presented in the center of the screen for 800–1,200 ms. Then, the magnitude (“100 Yuan”) of the delayed risky reward was presented for 1,000 ms. Next, after displaying a blank for 800–1,200 ms, the delay and the probability of a delayed risky reward was displayed for 2,000 ms. Then, after 500 ms, the choice (?100: 50) was presented, and its duration depended on the response of a participant. The cue “?100” represented the delayed risky option, and the cue “50” represented immediate certain option. A participant made a decision according to the subjective value of each option. If she/he considered that the subjective value of a delayed risky option was greater than 50 CNY, then pressed the left mouse button; if she/he considered the subjective value of a delayed risky option was less than 50 CNY, and then pressed the right mouse button. Finally, a blank screen was presented for 1,000 ms, and then the next trial started (Figure 1).

To control for the effects of physical characteristics of stimulus materials, all stimulus materials used the same color, font, and size, and all stimuli were randomly displayed. Considering the possible effects of location, the location of the delay and probability information was randomly assigned (up or down) on each trial and was counterbalanced across trials (Gui et al., 2016; Xia et al., 2017; Wang et al., 2019).

### Electroencephalography Recording and Analysis

Electroencephalography (EEG) recordings were continuously acquired at a 1,000-Hz sampling rate with a Neuroscan Synamp2 Amplifier, using an electrode cap with Ag/AgCl electrodes mounted according to the extended international 10–20 system. The EEG signals were amplified online (band pass, 0.05–100 Hz). All rows of electrode recordings were referenced online to the left mastoid, and then re-referenced offline to the average of the left and right mastoids. Electrode impedance was kept under 5 kΩ. Following electrode application, the participants sat in a comfortable chair located in a shielded room and were asked to fixate their gaze on the center of the computer display, which was located 1 m away from their eyes during the experiment.

EEG epochs of 1,000 ms (from −200 to 800 ms after the onset of delay and probability) were extracted offline, and the 200 ms pre-stimulus was defined as the baseline. Ocular artifacts were corrected. Trials contaminated by amplifier clipping, bursts of electromyographic activity or peak-to-peak deflection exceeding ±75 μV were excluded from further analysis. The remaining trials were baseline-corrected. Averaged ERPs were digitally filtered with a low-pass filter at 30 Hz. As a result, 39 (7.61), 39 (7.73), 38 (7.94), and 40 (8.29) trials were retained for ERP averaging for the SD + LP, SD + HP, LD + LP, and LD + HP conditions, respectively. There was no significant difference in the trial numbers between experimental conditions. Within-subject repeated-measures analyses of variance (ANOVAs) were used to analyze the ERP data using the factors delay (SD vs. LD) and probability (LP vs. HP). Behavioral and ERP data were statistically analyzed using SPSS (version 22; SPSS Inc., Chicago, IL, United States). A Greenhouse–Geisser correction for a violation of the sphericity assumption was applied when the degrees of freedom were more than one. The significance level was set at 0.05 for all analyses. To control for family wise error for multiple *t*-tests, *p* were Bonferroni corrected.

Based on visual inspection of the grand-average waveforms, four ERP components were analyzed. The frontal P200 was measured as the peak amplitude between 150 and 250 ms after stimulus onset at F3, Fz, and F4 (Polezzi et al., 2008; Molinaro and Carreiras, 2010; Gui et al., 2016). The N2 component was measured as the peak amplitude between 250 and 350 ms after stimulus onset at F3, Fz, and F4 (Gui et al., 2016; Xia et al., 2017; Wang et al., 2019). The P300 was measured as the mean amplitude between 280 and 420 ms after stimulus onset at P3, Pz, and P4 (Harris et al., 2013; Righi et al., 2014; Gui et al., 2016). The late positive potential (LPP) was measured as the mean amplitude between 500 and 700 ms after stimulus onset at P3, Pz, and P4 (Wu et al., 2012; Hua et al., 2014; Gui et al., 2016; Guo et al., 2018). ERP analyses were conducted using repeated-measures ANOVAs, with the factors delay (SD vs. LD) and probability (LP vs. HP).

## Results

### Behavioral Results

**Figure 2:**
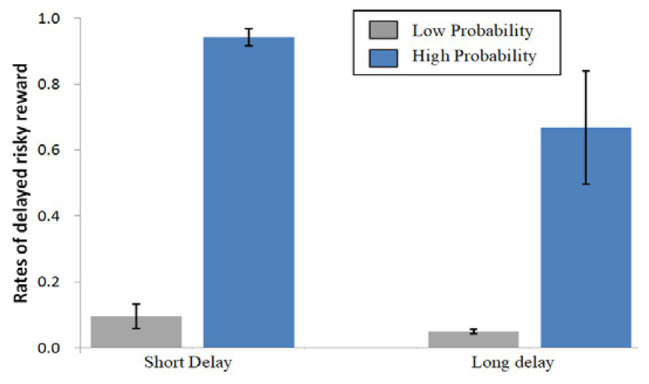
Mean percentage choices made for delayed risky options during the four conditions. Error bars denote standard error of the mean.

Figure 2 shows the percentages of delayed risky options during the four conditions. ANOVAs were conducted on the decisions made for delayed risky options using delay (SD vs. LD) and probability (LP vs. HP) as the within-participant factors. A significant main effect of delay was found [*F*(1,25) = 17.780, *p* < 0.001, *η_p_*^2^ = 0.416], indicating that short-delay options were selected more often than long-delay options. In comparison, a more significant main effect of probability on the percentage of delayed risky options was found [*F*(1,25) = 174.931, *p* < 0.001, *η_p_*^2^ = 0.875], indicating that more high-probability options were chosen than low-probability ones.

An interaction between delay and probability was found [*F*(1,25) = 9.846, *p* = 0.004, *η_p_*^2^ = 0.283]. For low probability, there was no simple effect of delay [*F*(1,25) = 2.173, *p* = 0.153, *η_p_*^2^ = 0.080]; for high probability, there was a significant simple effect of delay [*F*(1,25) = 16.217, *p* < 0.001, *η_p_*^2^ = 0.393]. There were significant simple effects of probability for the short-delay [*F*(1,25) = 344.317, *p* < 0.001, *η_p_*^2^ = 0.932] and long-delay [*F*(1,25) = 52.872, *p* < 0.001, *η_p_*^2^ = 0.679] conditions. This suggests that probability is given a higher decision weight than delay. Low probability crowded out the effect of delay discounting, while probability discounting and delay discounting coexisted for high-probability rewards.

The behavioral data showed that participants preferred short-delay rewards to long-delay rewards given the same probability, and preferred high-probability rewards to low-probability rewards given the same delay. These behavioral results demonstrated that participants clearly understood the experimental task (Gui et al., 2016; Wang et al., 2019).

Mean response times (RTs) of the decisions for the four conditions were 680 ± 187 ms (SD + LP), 604 ± 164 ms (SD + HP), 656 ± 184 ms (LD + LP), and 637 ± 163 ms (LD + HP), respectively. ANOVAs on the RTs of the decisions revealed a significant main effect of probability [*F*(1,25) = 9.060, *p* = 0.006, *η_p_*^2^ = 0.266] and interaction between probability and delay [*F*(1,25) = 5.297, *p* = 0.030, *η_p_*^2^ = 0.175], but no significant main effect of delay [*F*(1,25) = 0.184, *p* = 0.672, *η_p_*^2^ = 0.007].

There were no simple effects of delay for the low-probability [*F*(1,25) = 1.784, *p* = 0.194, *η_p_*^2^ = 0.067] and high-probability [*F*(1,25) = 4.063, *p* = 0.055, *η_p_*^2^ = 0.140] conditions. For short delay, there was a simple effect of probability [*F*(1,25) = 10.788, *p* = 0.003, *η_p_*^2^ = 0.301], but for long delay, there was no simple effect of probability [*F*(1,25) = 1.409, *p* = 0.246, *η_p_*^2^ = 0.053].

### ERP Results

#### P200

**Figure 3:**
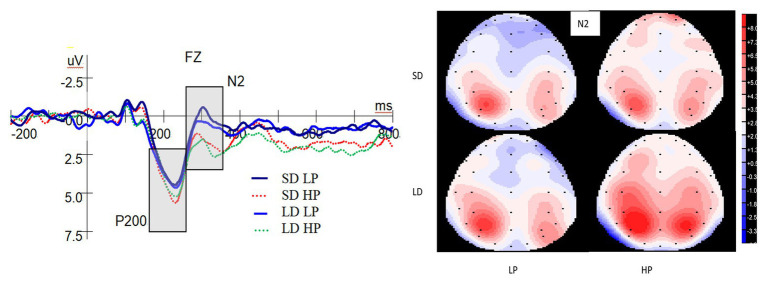
Grand-averaged event-related brain potential (ERP) waveforms at the Fz electrode for P200 and N2 and topographic maps (top view) for the N2. SD is short for short delay. LD is short for long delay. LP is short for low probability. HP is short for high probability.

Figure 3 reveals the ERP waveforms at the Fz electrode and topographic maps for the N2 in each of the four conditions. A significant main effect on P200 was observed for probability [*F*(1,25) = 6.195, *p* = 0.020, *η_p_*^2^ = 0.199], with larger P200 for high-probability compared to low-probability rewards. However, there was not significant main effect on P200 for delay [*F*(1,25) = 0.04, *p* = 0.953, *η_p_*^2^ = 0.000], and interaction between probability and delay [*F*(1,25) = 0.07, *p* = 0.933, *η_p_*^2^ = 0.000].

We applied ANOVAs on the latency of the P200. No significant main effect on the latency of the P200 for delay [*F*(1,25) = 0.363, *p* = 0.552, *η_p_*^2^ = 0.014] and probability [*F*(1,25) = 0.061, *p* = 0.807, *η_p_*^2^ = 0.002] were found, and there was no interaction between delay and probability [*F*(1,25) = 3.488, *p* = 0.074, *η_p_*^2^ = 0.122].

#### N2

As shown in Figure 3, there was a significant main effect on the N2 for probability [*F*(1,25) = 13.286, *p* = 0.001, *η_p_*^2^ = 0.347], with more pronounced N2 for low-probability compared to high-probability rewards. However, there was not significant main effect of delay [*F*(1,25) = 1.814, *p* = 0.190, *η_p_*^2^ = 0.068], and interaction between delay and probability [*F*(1,25) = 0.014, *p* = 0.907, *η_p_*^2^ = 0.001].

ANOVAs on the latency of N2 showed no significant main effects on the latency of N2 for delay [*F*(1,25) = 0.054, *p* = 0.818, *η_p_*^2^ = 0.002] and probability [*F*(1,25) = 0.985, *p* = 0.330, *η_p_*^2^ = 0.038], and there was no interaction between delay and probability [*F*(1,25) = 1.394, *p* = 0.249, *η_p_*^2^ = 0.053].

#### P300

**Figure 4:**
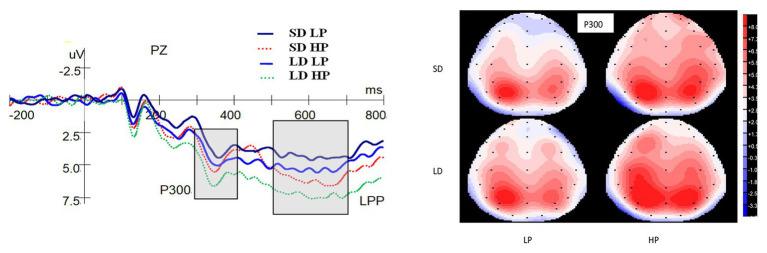
Grand-averaged ERP waveforms at the Pz electrode for P300 and LPP and topographic maps (top view) for P300. SD is short for short delay. LD is short for long delay. LP is short for low probability. HP is short for high probability.

Figure 4 shows the ERP waveforms at the Pz electrode and topographic maps for the P300. A significant main effect on the P300 was found for delay [*F*(1,25) = 8.373, *p* = 0.008, *η_p_*^2^ = 0.251], indicating that the P300 for long-delay conditions were more positive compared to short-delay conditions. There was also a significant main effect of probability on the P300 [*F*(1,25) = 10.020, *p* = 0.004, *η_p_*^2^ = 0.286], whereby high-probability rewards evoked significantly greater P300 than low-probability ones. There was no interaction between probability and delay [*F*(1,25) = 0.221, *p* = 0.642, *η_p_*^2^ = 0.009].

#### Late Positive Potential

As shown in Figure 4, there was a significant main effect on the LPP for probability [*F*(1,25) = 14.938, *p* = 0.001, *η_p_*^2^ = 0.374], whereby the amplitudes for high-probability rewards were significantly greater than those for low-probability ones. No significant main effect of delay on the LPP [*F*(1,25) = 3.807, *p* = 0.062, *η_p_*^2^ = 0.132], and no interaction between probability and delay [*F*(1,25) = 0.312, *p* = 0.581, *η_p_*^2^ = 0.012] were found.

#### Robustness Test

##### Correlation Analysis

In keeping with the goals of the study, a correlation test among these studied ERP components was performed. A strong correlation between P2 and N2 was observed (*r* = 0.687, *p* < 0.001). There was no significant correlation between P2 and P300 or LPP (*r* = 0.031, *p* = 0.754; *r* = 0.192, *p* = 0.051). A medium correlation between N2 and P300 (*r* = 0.206, *p* = 0.036) was found, and there was no correlation between N2 and LPP (*r* = 0.130, *p* = 0.188). There was no correlation between P300 and LPP (*r* = 0.178, *p* = 0.070).

##### N2 After Correcting for P200

In order to control for the effect of P200 on N2, we restructured a linear regression model for variance analysis of N2, given that ANOVA is considered to be a special case of linear regression. Statistical results showed that after correcting for P200, the main effect of probability was still significant (*T* = 2.758, *p* = 0.007), and the main effect of delay was not still significant (*T* = 1.860, *p* = 0.067). There was also no interaction between delay and probability (*T* = 0.107, *p* = 0.915).

##### ERP for Choice Type

In view of the polarization of decision behavior of the participants (Figure 2), to test whether neural processing of the stimuli differs between trials resulting in opposite choices, we averaged ERP separately for the two trial types defined by immediate certain vs. delayed risky option choice for all types of stimuli.

**Figure 5:**
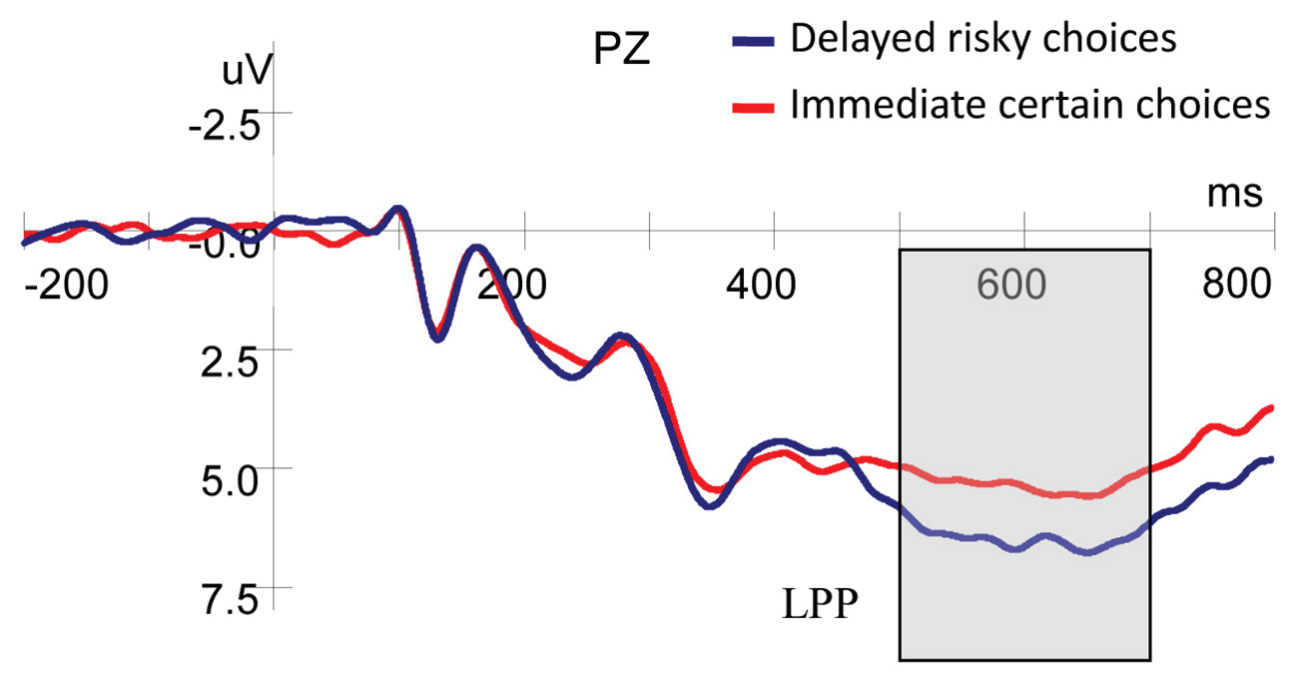
Grand-averaged ERP waveforms at the Pz electrode for choice type.

As shown in Figure 5, the results of ANOVA showed that the LPP amplitude evoked by stimuli (delay and probability) was larger for delayed risky choices compared with immediate certain choices [*F*(1,25) = 6.986, *p* = 0.014, *η_p_*^2^ = 0.218].

## Discussion

During delayed lotteries, subjective valuation of each option involves delay discounting and probability discounting. The present study focused on the combined effect of delay and probability in evaluating delayed risky rewards. Our behavioral results demonstrate that the effect of probability on delayed lottery choice is much more than that of delay. Our ERP results suggest that the P200, N2, P300, and LPP all reflect probability information, while only the parietal P300 is sensitive to delay information. Moreover, there was no interaction between delay and probability for these components. These results demonstrate distinct temporal dynamics for delay and probability processing during the evaluation of a delayed risky reward.

The frontal P200 was sensitive to probability, but not to delay. The amplitude of the P200 following high-probability rewards was more positive relative to that following low-probability ones. Existing research has shown that the P200 represents quick assessment and is sensitive to reward-related stimuli (Potts et al., 2004; Chen et al., 2009; Franken et al., 2010; Lau et al., 2013). Our findings suggest that the P200 represented superficial evaluation of stimuli and that only valuation of probability is distinguished. Our results are also consistent with the findings of Wang et al. (2019). They explored the neural responses to magnitude and probability of a risky reward. They found that the P200 was sensitive to probability, but no magnitude, with larger P200 amplitude following high-probability compared to low-probability rewards.

The N2, an early component following the P200, is characterized by a negative deflection occurring 250–350 ms (For reviews about N2, see Folstein and Van Petten, 2008). The N2 is considered to reflect the early evaluation of a reward value (Gehring, 2002; Azizian et al., 2006; He et al., 2012; Cherniawsky and Holroyd, 2013; Telpaz et al., 2015; Gui et al., 2016). The present study found that the N2 was sensitive to probability, but not to delay. This is consistent with the hypothesis that the N2 reflects superficial evaluation of rewards along the salient dimension and that information of other dimensions might not be encoded by the N2 (He et al., 2012; Wu et al., 2012; Cherniawsky and Holroyd, 2013; Gui et al., 2016). Previous studies showed that the N2 only represented reward valence when the stimuli involving reward valence and magnitude (Yeung and Sanfey, 2004; Sato et al., 2005) or involving reward valence and social distance (Yu and Zhou, 2006; Leng and Zhou, 2010). These studies provide support for our findings.

The effect of probability on N2 component in this study was similar to a pure probability discounting study (Wang et al., 2019). They applied a probability choice only paradigm and found that the N2 was sensitive to probability, with more pronounced N2 amplitude for low probability compared to high probability. Li et al. (2016) found that more negative N2 following low compared to high probabilities, and Yang and Zhang (2011) suggested that the N2 amplitude was more pronounced for high compared to low risk. Gui et al. (2016) and Xia et al. (2017) found that the N2 was sensitive to delay. Previous research has suggested that the N2 amplitude is more negative for unfavorable rewards relative to favorable ones (Goyer et al., 2008; Broyd et al., 2012; Umemoto et al., 2017; Wang et al., 2019). Because individuals prefer high-probability to low-probability rewards, the high-probability rewards are considered to be better than low-probability ones, given that all else is equal. As such, we found that the N2 was more negative for low-probability relative to high-probability rewards.

In contrast to the N2, the P300 was sensitive to both probability and delay in the present study. The P300, which is generally considered to relate to the allocation of attentional resources, is sensitive to controlled process of evaluation (Wu and Zhou, 2009; Ferrari et al., 2011; Righi et al., 2012; Pfabigan et al., 2014; Guo et al., 2018). Previous studies have found that the P300 could represent both reward valence and magnitude in gambling tasks, indicating more pronounced P300 following larger outcomes and positive compared to negative outcomes (Wu and Zhou, 2009; Leng and Zhou, 2010; Wu et al., 2012). The larger P300 amplitude following high-probability rewards suggests that the P300 can differentiate favorable outcomes from unfavorable outcomes during reward evaluation (Wu and Zhou, 2009; Wu et al., 2012; Gui et al., 2016). However, the finding of larger P300 following long-delay compared to short-delay rewards cannot be explained by the favorability evaluation hypothesis. One possible explanation for this might be the modulation of the P300 by the magnitude. Previous studies have showed that the P300 represents reward magnitude, with a more pronounced amplitude for a larger reward amount, regardless of whether the P300 is sensitive to reward valence (Yeung and Sanfey, 2004; Leng and Zhou, 2010). Furthermore, the studies on pure delay or probability discounting also found that the P300 was sensitive to delay or probability. Gui et al. (2016) found that the P300 was sensitive to delay, with larger P300 amplitude for short delay compared to long delay. Li et al. (2016) found that high probabilities evoked more positive P3 than low probabilities.

Unlike the P300, the LPP only represented probability information in the present study. The posterior LPP has been found to be implicated in evaluative processing (Ferrari et al., 2011; Righi et al., 2012). A large body of research suggested that positive and negative stimuli evoked a larger LPP than neutral stimuli (Schupp et al., 2000; Hua et al., 2014; Guo et al., 2018). Wu et al. (2012) suggested that the LPP only represented social comparison, not valence. Wang et al. (2019) reported that the LPP was sensitive to probability and insensitive to magnitude when evaluating risky rewards, with the amplitude of the LPP larger for high-probability rewards. Moreover, the LPP has been shown to be largest in response to stimuli with the greatest motivational relevance (Schupp et al., 2000; Wu et al., 2012). In our study, high-probability rewards were of great motivational importance, because they increased the chance of a participant making a large gain and affected their subsequent response. Therefore, the LPP reflected the probability process, with a larger amplitude following high-probability rewards compared to low-probability ones.

Our behavioral data revealed a significant interaction between delay and probability when making a delayed risky decision, supporting multiplicative discounting models. However, there was no interaction between delay and probability based on our ERP data, which supports additive discounting models. Thus, the results from the behavioral and the neural data seem to be at odds. The reason for this discrepancy may be that the behavioral and the ERP data measure responses at different stages of the decision making. At the early stage, the human brain encodes delay and probability, respectively; by contrast, at the later stage of selection, the brain engages in more complex processing activities, and delay and probability information begin to interact (Kahneman, 2011).

Moreover, previous behavioral studies suggest that the probability weighting is much larger than the delay weighting during the evaluation of a delayed risky outcome (Shavit and Rosenboim, 2015; Vanderveldt et al., 2015; Weatherly et al., 2015). Our finding in which probability has a prolonged effect on the evaluation of delayed risky rewards compared to delay is consistent with these studies.

In order to validate the conclusion of distinct temporal dynamics for delay and probability processing when combined discounting, it would be more interesting to explore neural responses to more delay and probability combinations. The limitation of this study is that it only explores the combined discounting of four delay and probability combinations, and these task parameters may affect our conclusion. Therefore, future studies can further explore the neural responses to more delay and probability combinations.

## Conclusion

To sum up, this study investigated neural correlates underlying the combined discounting of both delay and probability during the evaluation of a delayed risky reward. The findings of this study suggest that there are different responses to probability and delay when evaluating a delayed risky reward. First, at the early stage, the P200 and the N2, which represent spontaneous, effortless, and unintentional processes, were modulated by probability, but were insensitive to delay. Additionally, at the elaborative evaluation stage, the P300 component reflected both probability and delay. Finally, at the reappraisal stage, the LPP was only sensitive to probability, but was insensitive to delay. These results suggest that probability information has a prolonged effect on the evaluation of delayed risky rewards compared to delay information. These findings provide neurophysiological evidence for the combined discounting of delay and probability.

## Data Availability Statement

The raw data supporting the conclusions of this article will be made available by the authors, without undue reservation.

## Ethics Statement

The studies involving human participants were reviewed and approved by the Ethics Committee of the School of Economics, Shandong University, China. The patients/participants provided their written informed consent to participate in this study.

## Author Contributions

GW and JL designed this study. CZ and SL implemented the experimental protocols and collected the data. SL and GW analyzed the data. GW and JL wrote the manuscript. All authors contributed to the article and approved the submitted version.

### Conflict of Interest

The authors declare that the research was conducted in the absence of any commercial or financial relationships that could be construed as a potential conflict of interest.
